# Hydrocele, Cured by Injections with a Simple Apparatus

**Published:** 1802-08-01

**Authors:** William Robertson

**Affiliations:** one of the Surgeons to the Kelso Dispensary


### *Hydrocele, cured by Injections with a simple Apparatus, by* William Robertson, *one of the Surgeons to the Kelso Dispensary.*

YOUR Monthly Medical Journal muſt ever be eſteemed valuable to all thoſe who are well-wiſhers to the healing Art, for the early information of the improvements of Phyſick and Surgery it diffuſes every where, beſides the ready acceſs it affords to all thoſe who are diſpoſed to communicate to the Public ſuch improvements they have found uſeful in the courſe of their practice. Without ſuch a vehicle of conveyance, many important diſcoveries would in a great meaſure be loſt, unleſs to the individual diſcoverer.

Induced by theſe conſiderations, I take the liberty of tranſmitting to you the following two Caſes of Hydrocele, cured by Injections. Not that I claim any merit in diſcovering the principle, but in deſcribing a cheap apparatus, and in explaining the mode of performing the operation with facility. If it ſhould merit an inſertion in your laudable Publication, I ſhall conſider any trouble I have beſtowed in making the diſcovery gratefully compenſated.

Case I. James P. æt. 63, by trade a wheelwright, called upon me to take my advice with regard to a ſwelling he had in the ſcrotum, which had been of ſome years duration, but always upon the increaſe, till it had arrived to ſuch a ſize that it was become quite an incumbrance, and prevented him in a great meaſure from following his daily employment. He attributed the origin of it to a ſtroke he got on that part at ſome diſtant period. Upon a careful examination of the tumour, I found it to be a collection of water in the tunica vaginalis teſtis. He was then informed, the only chance he had of being relieved from his complaint, would be by undergoing an operation, and that it might be done in two ways. The firſt was the palliative meaſure, by drawing off the water with the trocar; and it was ſo ſimple, that he would be able follow his employment in the courſe of a day or two after; at the ſame time he was acquainted, that he would be liable to a return of the diſorder after a lapſe of time. The other mode of performing the operation would prove a complete cure; but it would be a little more painful, and confine him a few weeks from following his employment. He preferred the former, becauſe he could not think of being prevented from working. Having introduced the trocar into the ſcrotum and through the tunica vaginalis in the common way, I drew off a pound and two or three ounces of an uriniferous coloured fluid; found the tefticle quite in a found ftate; he was relieved from his burden, and able to follow his employment a very little while after. In the courfe of a month he began to obferve the fwelling again; it continued increafing gradually for the fpace of fix months, till it was nearly as large as at firft, and became quite an incumbrance. I was again confulted and gave it as my opinion, that the beft thing he could do was to undergo an operation for the radical cure; mentioning to him there were two ways of performing it; the one was by laying the tumour open nearly the whole length, and evacuating the water; the other was done by drawing off the water by the trocar and canula, as had been done already, and injecting a certain proportion of wine and water. The latter he made choice of, as being lefs formidable. The day being appointed for performing the operation, but not being in poffeffion of the apparatus generally made ufe of for that purpofe, I thought of the following device: Having procured a bladder that held about a pound and a half, I mounted it on the end of the canula, exactly in a fimilar way as a bladder is mounted on a glyfter-pipe; it was then rolled firmly up, fo that it might occupy as little fpace as poffible, for fear of preventing the flow of the water. This being done, the patient was placed on a feat of a convenient height; I then introduced the trocar and canula, and drew off about 18 ounces of an urine-coloured fluid. It was received into a *strouped* decanter; the height the water arofe to in the decanter was exactly marked with a piece of wet paper pafted on the infide; it was then emptied out, making an affiftant hold the canula with the bladder in the fcrotum; two glaffes of wine with one of water were poured into the decanter, alternately, until it arofe to the paper pafted on the fide of it; the bladder was then *unloosed*, and the affiftant defired to hold the mouth of it open till all the wine and water was poured in; it was then firmly tied with a piece of *knitting* round the mouth, and the whole of the fluid was injected into the fcrotum, which made it fwell exactly to the fame fize it was before the water was drawn off; I then caufed the bladder to be tied clofe to the canula with a piece of *knitting*, to prevent its return, (having previoufly put my watch upon the table). At firft he complained of a great degree of coldnefs, with a kind of thrilling pain going up the fpermatic cord. After being confined there for eight minutes, he began to feel a degree of warmth, which extended to the loins; but to fecure the fuccefs of the operation, it was allowed to remain four minutes longer; the *knitting* at both parts was unloofed, and all the liquor was emptied into a bafon. A fufpenfary bandage was applied to the fcrotum, and he was ordered dered to be carried to bed, and take an anodyne draught. In the courfe of twenty-four hours the inflammation came on, and continued to increafe till it was as large as it was before the water was drawn off, accompanied with pain and hardnefs. It continued in that ftate for about ten days, during which time he took three dofes of falts and manna, and kept a bread and milk poultice over it. The wound made by the trocar continued to difcharge a fmall quantity of matter for fome time after this period; the inflammation and pain began to abate, and by continuing the above mode of treatment, in the courfe of fixteen days from the operation, he was able to walk about and do a little work. A degree of hardnefs remained in the body of the tefticle for fome time; at laft it began to diminifh gradually till it became the fize of the other. It is now three months fince the operation was performed, and he is quite well and following his daily employment, without the fmalleft figns of the complaint returning.

Case II. I was requefted to vifit George R. aged fixty, a common day labourer. He had been afflicted with a fwelling in the fcrotum, of fome years ftanding; he was tapped, which relieved him from his burden for fome time, but it returned again, and was become now larger than ever. I took Mr. Fleming, a navy furgeon, an acquaintance of mine, along with me to examine it; we found it to be an hydrocele of the tunica vaginalis teftis, and advifed him to have the operation for the radical cure performed, which he at firft had a great averfion to. Having pointed out the propriety of the meafure in very ftrong terms, he at laft agreed to fubmit to it; but unfortunately, being fo fituated, we could not procure any Port wine, and being unwilling to delay the operation after having brought his mind to the refolution, it was at laft fuggefted to inject certain proportions of fpirits and water in place of the wine; this being refolved upon, we procured fome fpirit called whifkey, which is the common liquor that is drank in the country. The patient being conveniently feated, the trocar and canula with the bladder mounted on it, were introduced into the tunica vaginalis teftis; about a pound of an uriniferous coloured fluid was drawn off into a decanter, and the height where it arofe to was marked with paper; it was then emptied out, and a glafs of whifkey and another of water were poured into it, alternately, till it reached the part of the decanter marked with paper; the bladder was loofened, and the whifkey and water were emptied into it from the decanter, and the reft of the operation was finifhed exactly in the fame way as is mentioned in Cafe I. After remaining about the fpace of a quarter of an hour, he began to feel fome degree of pain; and to enfure the fuccefs of the operation it was confined for three or four minutes longer, then it was withdrawn. But he never felt the fame degree of cold fucceeded by heat, nor pain, as was produced by the wine. The fufpenfary bandage being applied to the fcrotum, he was orderded to bed. The inflammation began to make its appearance the day after the operation, and continued to increafe to nearly the fize it was before the water was drawn off. The wound made by the trocar oozed a little matter; was ordered a cooling cathartic compofed of an ounce and a half of falts, and half an ounce of manna, and to apply an emollient poultice over it, which was repeated frequently for a few days; but the inflammation and pain became moderate, and it was difcontinued, neither was it thought neceffary to repeat the cooling cathartic again; he kept his bed for about ten days, after which period the fwelling gradually fubfided, together with the hardnefs of the tefticle; he is now following his ufual employment, and no figns of the diforder returning, though a confiderable time has elapfed fince.

The proximate caufe of this difeafe is either a morbid fecretion of the fecreting veffels or a deficiency in the office of the lymphatics in that part. The fuccefs of the cure depends on injecting a proper proportion of a ftimulating liquor exactly equal to the quantity of collected fluid drawn off, and confining it for fuch a length of time as to produce a gentle inflammation over the furface of the tunica vaginalis teftis; in confequence of this an adhefion takes place betwixt it and the body of the tefticle, and obliterates the cavity fo as to prevent the return of the diforder. This operation can be performed with great exactnefs and facility, by mounting the bladder on the canula, fo as to form an injection bag and pipe; and the patient fuffers no pain from the inftrument but on its firft introduction, the management of which has already been fully explained in the above two cafes.

CRITICAL

